# Nurses’ Experiences With Artificial Intelligence and the Association Between Clinical Decision‐Making and Missed Nursing Care: A Convergent Mixed‐Methods Study

**DOI:** 10.1155/jonm/5948812

**Published:** 2026-10-11

**Authors:** Faisal Khalaf Alanazi, Ahmed Farghaly Tawfik, Shimaa Abdelfattah Mahgoub, Ibrahim Naif Alenezi, Mohamed Hussein Ramadan Atta, Ayman Mohamed El-Ashry, Enas Mohamed Lotfy, Alshaimaa Yehia Mohammed Yehia

**Affiliations:** ^1^ Medical Surgical Nursing Department College of Nursing Northern Border University Arar Saudi Arabia nbu.edu.sa; ^2^ Department of Public Health Nursing College of Nursing Northern Border University Arar Saudi Arabia nbu.edu.sa; ^3^ Nursing Administration Department Faculty of Nursing Beni-Suef University Beni-Suef Egypt bsu.edu.eg; ^4^ Nursing Department College of Applied Medical Sciences Prince Sattam Bin Abdulaziz University Wadi Addawasir 18734 Saudi Arabia psau.edu.sa; ^5^ Faculty of Nursing Alexandria University 21511 Alexandria Egypt alexu.edu.eg; ^6^ Psychiatric and Mental Health Nursing Department Faculty of Nursing Alexandria University Alexandria Egypt alexu.edu.eg; ^7^ Department of Public Health Nursing College of Nursing Northern Border University Arar Saudi Arabia nbu.edu.sa

**Keywords:** artificial intelligence, clinical decision-making, missed nursing care, mixed-methods

## Abstract

**Introduction:**

Artificial intelligence (AI) is increasingly being incorporated into clinical nursing practice, but evidence remains limited on how nurses experience AI‐supported systems and how decision‐making orientation relates to missed nursing care (MNC).

**Aim:**

To explore nurses’ experiences with AI‐supported clinical decision support (CDS) and examine associations among clinical decision‐making orientation, professional characteristics, and MNC.

**Design:**

A convergent parallel mixed‐methods study.

**Methods:**

The study was conducted in six hospitals in Beni Suef Governorate, Egypt. The quantitative sample included 210 intensive care unit (ICU) nurses who completed the Nursing Decision‐Making Instrument–Revised 2014 (NDMI‐R) and the Missed Intensive Nursing Care Scale (MINCS). Fourteen nurses completed semistructured interviews, and all transcripts were included in the thematic analysis.

**Results:**

NDMI‐R scores showed a small negative bivariate correlation with MNC (*r* = −0.178, *p* < 0.01), but the adjusted association was not statistically significant (*β* = −0.124, *p* = 0.063). The regression model explained 16.9% of the variance in MNC (adjusted *R* ^2^ = 14.5%). Years of experience (*β* = 0.198, *p* = 0.004) and bachelor’s degree or above compared with a technical nursing diploma (*β* = −0.255, *p* < 0.001) were significant predictors. The other education contrast, AI training/support and MISSCARE Reasons were not statistically significant. In interviews, nurses described AI‐related tools as potentially helpful for alerts, information review, workflow, and communication while also raising concerns about trust, overreliance, empathy, and training.

**Conclusion:**

Nurses perceived potential benefits from AI‐supported tools, but the quantitative findings did not establish an independent effect of AI or a statistically significant adjusted association between decision‐making orientation and MNC. These findings support cautious, nurse‐centered implementation and further longitudinal evaluation.

**Implications for Nursing Management:**

Managers should pair any AI‐supported CDS implementation with role‐specific training, clear accountability, usability monitoring, and safeguards that preserve clinical judgment and humanistic care.

## 1. Introduction

Missed nursing care (MNC) refers to necessary nursing activities that are omitted, delayed, or only partly completed. It remains a persistent concern because it is associated with poorer patient and organizational outcomes, including falls, infections, complications, longer hospital stays, and lower patient satisfaction [1, 2]. Staffing, material resources, communication, and the broader safety culture can all shape whether care is completed as intended [3, 4].

Artificial intelligence (AI) is becoming increasingly visible in nursing practice [5, 6]. In this study, AI‐related clinical decision support (CDS) refers specifically to automated, computational systems with the following characteristics: (1) predictive analytics or algorithms that generate patient‐risk scores or clinical alerts; (2) adaptive functions that modify recommendations based on new clinical data; or (3) algorithmic decision‐support tools that synthesize complex clinical information and present structured recommendations at the point of care [7]. Examples include sepsis prediction algorithms, ventilator weaning recommendations, medication interaction alerts, or AI‐enabled documentation systems that synthesize clinical notes [6]. Excluded from this definition are conventional electronic health record (EHR) functions (e.g., standard drop‐down menus, static templates), barcode medication administration systems, and passive safety devices (e.g., smart pumps with fixed guardrails) unless these systems incorporated predictive, adaptive, or algorithmic components. The operational value of these tools depends on practical implementation factors such as usability, workflow integration, cognitive burden, explainability of recommendations, and clinician trust [7–9]. These implementation characteristics are essential to understanding how algorithmic systems function within complex clinical environments rather than being inherent properties of the algorithms themselves.

Three complementary frameworks informed the interpretation of this study. The Donabedian structure–process–outcome model places AI‐supported CDS within the organizational resources that may shape care processes and outcomes. The Systems Engineering Initiative for Patient Safety (SEIPS) framework emphasizes the interaction among technology, tasks, people, the environment, and organizational conditions [9]. The Consolidated Framework for Implementation Research (CFIR) helps situate nurses’ experiences within the characteristics of the intervention, the inner setting, individual factors, and the implementation process [10]. These frameworks provided context for interpretation; the study did not directly measure every construct within them.

A key distinction is that clinical decision‐making orientation is not the same as decision quality. The Nursing Decision‐Making Instrument–Revised 2014 (NDMI‐R 2014) identifies whether nurses tend toward a more analytic or intuitive orientation. Higher scores indicate a more intuitive orientation; they do not indicate that decisions are better or more accurate. MNC and reasons for missed care were assessed with the Missed Intensive Nursing Care Scale (MINCS).

Previous work has examined AI adoption, nursing decision support, and MNC separately, but less is known about how ICU nurses describe AI‐related tools alongside measured decision‐making orientation and missed care in the Egyptian context. A convergent mixed‐methods design can bring these perspectives together without assuming that one variable causes another.

Accordingly, this study aimed to explore nurses’ experiences with AI‐supported CDS and to examine associations among clinical decision‐making orientation, professional characteristics, and MNC. The study used self‐report questionnaires and semistructured interviews. It did not include objective workload measures, safety‐culture measures, system‐use logs, or observational data, and its cross‐sectional design does not support causal claims about the effect of AI.

## 2. Methods

### 2.1. Study Design and Setting

The current study has a convergence parallel design with the application of a mixed‐methods approach. In this type of design, both qualitative and quantitative data were gathered at the same time but analyzed separately. The results of both were then compared and put together in the interpretation stage. Such a convergent parallel design application was used to provide a holistic perspective of the research problem given that it combined the knowledge of both the qualitative and quantitative views. During the research, the COREQ guidelines of qualitative studies and the STROBE checklist of cross‐sectional studies were followed to guarantee adherence to rigor and adherence to the specified criteria. The research was carried out in six hospitals in Beni Suef Governorate, which is in Middle Egypt. At the interpretation stage, the separately analyzed strands were integrated by matching each adjusted quantitative finding with relevant qualitative themes, classifying the relationship as convergence, complementarity, or expansion, and retaining findings that were not directly explained rather than forcing agreement. These linkages were summarized in a joint display to support explicit meta‐inferences.

### 2.2. Participants and Sampling

Eligible participants were registered nurses (RNs) providing direct care in intensive care units (ICUs), with at least 2 years of full‐time ICU‐specific experience and self‐reported exposure to an AI‐related clinical tool. During eligibility screening, participants described exposure to functions such as predictive alerts, risk scoring, algorithm‐supported recommendations, or adaptive decision support. Conventional EHR functions, barcode medication administration, and standard smart‐pump safeguards were not classified as AI unless the participant described a predictive, adaptive, or algorithmic component.

Exposure was determined by participant self‐report. The study did not obtain system‐use logs, vendor names, software versions, or technical specifications, and it did not use a standardized daily or weekly frequency measure. Interview accounts therefore supported only broad descriptions of routine or regular, intermittent, or minimal exposure. These descriptions should not be interpreted as numerical exposure estimates.

Nurses were excluded if they had less than 2 years of ICU‐specific experience, worked outside an ICU, or were temporary or part‐time staff who were not consistently involved in ICU practice. Total nursing experience and ICU‐specific experience were recorded separately. Convenience sampling was used for the quantitative phase. Purposive sampling was used for the qualitative phase to recruit eligible nurses with direct experience of AI‐related tools.

The quantitative sample size was estimated using G∗Power 3.1.4 for linear multiple regression [11]. With *α* = 0.01, power = 0.80, a medium effect size (*f* ^2^ = 0.15), and 17 candidate predictors, the minimum required sample was 195. The target was increased to 220 to allow for incomplete responses. Ten questionnaires were incomplete, leaving 210 for analysis and a response rate of 95.5%.

For the qualitative phase, 20 eligible nurses were invited and 14 completed an interview. Recruitment ended when successive interviews produced no new codes, categories, or themes [12]. All 14 completed transcripts were included in the final thematic analysis.

### 2.3. Instruments

#### 2.3.1. Part I: Qualitative Interview Guide

A semistructured interview guide was developed to explore nurses’ experiences of AI‐related tools in clinical decision‐making and MNC. Its 10 questions were informed by previous studies of healthcare professionals’ views of AI and its use in healthcare [13, 14]. Questions addressed perceived usefulness and ease of use, trust, possible support for clinical judgment, communication, readiness for change, barriers to use, and concerns about AI in nursing practice.

The guide was pilot‐tested with a small group of nurses to improve clarity and relevance. Questions were then phrased to invite descriptions rather than assume that AI had produced an effect. For example, nurses were asked how using an AI‐related tool had shaped their confidence or independence in a specific situation, followed by prompts such as “Can you give an example?” The final guide was used in individual interviews and is provided in the article’s appendix.

#### 2.3.2. Part II: Quantitative Data Collection Questionnaires

A structured questionnaire with three sections comprising (1) a demographic information form, (2) NDMI‐R 2014, and (3) MINCS was used to collect quantitative data. This format guaranteed the overall data gathering on the background of participants, the process of decision‐making, and the cases of nursing care omission in the ICU.

#### 2.3.3. Demographic Information Form

The form was used to collect data on age, gender, place of residence, household income, marital status, education level, department, total years of nursing experience, years of ICU‐specific experience, and previous support or training in the use of AI tools.

#### 2.3.4. The MINCS

MINCS, developed by Yang et al. [15], assesses missed care in intensive care settings. The present study used Part A and Part C of the scale. Part A contains 38 items across five domains: physiology (5 items), safety (13 items), belongingness (7 items), esteem (10 items), and cognition (3 items). It measures how often nursing activities are missed. Part C contains 23 items covering reasons for missed care: labor resources (4 items), material resources (4 items), communication factors (4 items), and managerial factors (11 items).

Part A items are rated on a five‐point scale from 1 (*never missed*) to 5 (*always missed*), with higher scores indicating more frequent missed care. Part C items are rated from 1 (*not a reason*) to 4 (*a significant reason*), with higher scores indicating that the listed factors are viewed as more important reasons for missed care. All frequency‐of‐missed‐care analyses reported here use Part A, whereas analyses of reasons for missed care use Part C.

The original scale showed good internal consistency (Cronbach’s *α* = 0.85) and a Guttman split‐half coefficient of 0.72. For this study, the scale was translated into Arabic and showed strong internal consistency (Cronbach’s *α* = 0.90).

#### 2.3.5. NDMI‐R 2014

NDMI was developed to describe whether nurses tend to use a more analytic or intuitive approach when making clinical decisions. The revised 2014 version (NDMI‐R 2014) contains 24 items representing four stages: data collection; data processing and problem identification; action planning; and implementation, monitoring, and evaluation [16, 17].

Items are rated on a five‐point scale from 1 (*never or almost never*) to 5 (*almost always or always*). Higher total scores indicate a more intuitive decision‐making orientation, whereas lower scores indicate a more analytic orientation. The NDMI‐R does not measure the quality, accuracy, or effectiveness of a nurse’s decisions; therefore, a higher score should not be interpreted as better decision‐making.

Previous work has reported Cronbach’s *α* values of 0.85 for the analytic subscale and 0.83 for the intuitive subscale [17]. In the present study, the Arabic NDMI‐R 2014 had strong internal consistency (Cronbach’s *α* = 0.935 for the total scale).

### 2.4. Tool Preparation and Pilot Testing

The MINCS and NDMI‐R 2014 were translated into Arabic using forward and back translation. Two bilingual researchers completed the initial translations, and independent bilingual specialists back‐translated the instruments into English. Differences were discussed until linguistic equivalence was reached. Five nursing experts assessed face validity. A pilot study with 20 nurses confirmed that the items were clear and relevant. No further wording changes were required. In the present sample, Cronbach’s *α* was 0.90 for the MINCS and 0.935 for the NDMI‐R 2014.

### 2.5. Data Collection

For the qualitative phase, 20 eligible nurses were invited to participate, and 14 completed an interview. Interviews were conducted in Arabic in quiet rooms within the hospitals, audio‐recorded with consent, and transcribed verbatim. For publication, selected quotations were translated into English and checked against the Arabic transcripts by a bilingual member of the research team. All 14 completed transcripts were analyzed. For the quantitative phase, eligible nurses completed self‐administered questionnaires. Written informed consent was obtained, and completed questionnaires were returned in sealed envelopes. Data were collected from early December 2024 through the end of February 2025.

### 2.6. Ethical Considerations

The Research Ethics Committee of Beni Suef University, Egypt, gave its approval to this study (Reference No. FNBSURECP/2811202416). The study was conducted in accordance with the ethical standards of the Helsinki Declaration, whereby all the participants were provided with an elaborate explanation of the study goals and methodologies. During the process, voluntary participation, anonymity, and confidentiality were highly ensured. Another issue that was made clear to the participants was their right to withdraw at any point without any adverse effects.

### 2.7. Data Analysis

#### 2.7.1. Qualitative Data Analysis

Interview data were analyzed using Braun and Clarke’s six‐phase approach to thematic analysis [18]. Two nurse‐researchers with experience in qualitative nursing research first read the Arabic transcripts independently, generated initial codes, and grouped related codes into candidate themes. They then compared their coding and discussed differences until they reached consensus. The wider research team reviewed the developing themes against the transcripts and agreed on the final names and definitions.

Coding decisions and changes to the thematic structure were documented to maintain an audit trail. Analysis was completed manually; rigor came from independent coding, comparison, consensus, team review, and close attention to participants’ accounts rather than from the choice of software. Data saturation was judged to have occurred when successive interviews produced no new codes, categories, or themes.

All 14 completed interviews met the inclusion and consent requirements and contained material relevant to AI‐related tools, clinical decision‐making, or MNC. No completed transcript was excluded. Interviews were conducted and coded in Arabic. Quotations selected for the manuscript were translated into English, checked against the original Arabic wording, and reviewed for consistency of meaning.

#### 2.7.2. Quantitative Statistical Analysis

Data were checked before entry and analyzed using IBM SPSS Statistics, Version 25.0. Descriptive statistics summarized participant characteristics and study variables. Pearson correlation coefficients were used for bivariate associations. Statistical significance was set at *p* < 0.05; *p* ≤ 0.01 was described as highly significant. Before the final analyses, all scale items and composite scores were rechecked for coding consistency. Item coding was standardized, composite scores were recalculated, and the correlation and regression analyses were rerun using the corrected final dataset.

The final analyses reported in this manuscript used the original, non‐Winsorized observations. The earlier Winsorization description and the reference to a supplementary sensitivity table were removed because Winsorization was not retained in the final model. Normality and homoscedasticity were assessed using distributional checks and residual plots. Multicollinearity was examined with tolerance and variance inflation factor (VIF) values.

Multiple linear regression was used to examine adjusted associations with MNC. The predictors were years of experience, educational level, support or training in the use of AI tools, NDMI‐R score, and the MISSCARE Reasons composite. Educational level was dummy‐coded with technical nursing diploma (TND) as the reference category, producing two contrasts: secondary nursing diploma (SND) versus TND, and bachelor’s degree or above versus TND. The training/support indicator was coded as 1 = did not receive training or support and 0 = received training or support. All predictors were entered simultaneously.

The MISSCARE Reasons composite (MINCS Part C) was included as a contextual covariate because labor, material, communication, and managerial constraints may be related to missed care. Its adjusted coefficient was interpreted as an independent linear association within the simultaneous model, not as a test of whether contextual barriers matter clinically. A hierarchical analysis was not added because no ordering of predictor blocks or incremental‐variance hypothesis had been specified in advance.

## 3. Results

### 3.1. Quantitative Data Results

Table 1 shows that participants had a mean age of 25.8 years; 89.5% were women and 64.3% lived in rural areas. More than half held a TND (54.3%), and 81.4% reported receiving some support or training in the use of AI tools.

**TABLE 1:** Sociodemographic and work‐related characteristics of participants (*n* = 210).

Variable	Category	Frequency	Percentage
Age	< 25	76	36.2
	25–35	125	59.5
	35+	9	4.3
	Mean ± SD	25.8 ± 4.9	

Gender	Male	22	10.5
	Female	188	89.5

Place of residence	Urban	75	35.7
	Rural	135	64.3

Family income	Not‐Enough	34	16.2
	Enough	133	63.3
	Enough and save	43	20.5

Marital status	Not Married	104	49.5
	Married	106	50.5

Experience	2–4	81	38.6
	5–10	100	47.6
	10+	29	13.8
	Mean ± SD	5.6 ± 3.1	
Educational level	SND	13	6.2
	TND	114	54.3
	Bachelor and above	83	39.5

Shift type	Day	107	51.0
	Night	21	10.0
	Rotating	82	39.0
	Total	210	100.0

Receiving support/training for using AI tools	Yes	171	81.4
	No	39	18.6
	Total	210	100.0

Abbreviations: MD, master’s degree; SND, secondary nursing diploma; TND, technical nursing diploma.

Table 2 presents the correlations among the three composite measures. NDMI‐R score was weakly and negatively correlated with MNC (*r* = −0.178, *p* < 0.01). MISSCARE Reasons was weakly correlated with NDMI‐R score (*r* = −0.113) and with MNC (*r* = −0.081); neither correlation was statistically significant. These findings describe bivariate associations and do not indicate causal effects or decision quality.

**TABLE 2:** Descriptive statistics and pairwise correlations of the study variables (*n* = 210).

	1	2	3
1. Decision‐making			
2. MISSCARE	−0.178^∗∗^		
3. MISSCARE Reasons	−0.113	−0.081	
Mean ± SD	93.5 ± 16.4	80.7 ± 39.8	62.8 ± 16.9

^∗∗^Correlation is significant at the 0.01 level (2‐tailed). Higher NDMI‐R (decision‐making) scores indicate a more intuitive decision‐making orientation; higher MINCS Part A (MISSCARE) scores indicate more frequent missed nursing care; and higher MINCS Part C (MISSCARE Reasons) scores indicate greater perceived importance of reasons for missed care.

The multiple linear regression model was statistically significant (*F* = 6.90, *p* < 0.001) and explained 16.9% of the variance in MNC (*R* ^2^ = 0.169; adjusted *R* ^2^ = 0.145). Years of experience was positively associated with MNC (*B* = 2.546, *β* = 0.198, *p* = 0.004). Compared with nurses holding a TND, those with a bachelor’s degree or above had lower MNC scores (*B* = −20.771, *β* = −0.255, *p* < 0.001), whereas the SND contrast was not significant (*p* = 0.107). The indicators for not receiving AI training/support (*p* = 0.082), NDMI‐R score (*β* = −0.124, *p* = 0.063), and MISSCARE Reasons (*β* = −0.126, *p* = 0.057) were not statistically significant. Tolerance values ranged from 0.883 to 0.939 and VIF values from 1.065 to 1.133, indicating no evidence of problematic multicollinearity.

Table 3 presents the full multiple linear regression model predicting MNC, including unstandardized and standardized coefficients, 95% confidence intervals, and collinearity statistics for all predictors.

**TABLE 3:** Regression analysis predicting missed nursing care (MISSCARE).

Model		Unstandardized coefficients		Standardized coefficients	*t*	Sig.	95.0% confidence interval for *B*		Collinearity statistics	
		*B*	Std. error	*β*			Lower bound	Upper bound	Tolerance	VIF
1	(Constant)	134.782	21.122		6.381	< 0.001	93.135	176.429		
	Years of experience	2.546	0.864	0.198	2.946	0.004	0.842	4.249	0.907	1.103
	Educational level (TND): SND	17.771	10.968	0.108	1.620	0.107	−3.854	39.397	0.927	1.079
	Educational level (TND): Bachelor	−20.771	5.539	−0.255	−3.750	< 0.001	−31.693	−9.850	0.883	1.133
	Not receiving support/training for using AI tools	−11.935	6.827	−0.117	−1.748	0.082	−25.397	1.526	0.919	1.089
	Clinical decision‐making	−0.302	0.162	−0.124	−1.868	0.063	−0.621	0.017	0.923	1.083
	MISSCARE Reasons	−0.298	0.156	−0.126	−1.911	0.057	−0.604	0.009	0.939	1.065

*Note:* Dependent variable: MISSCARE, Model = ANOVA, *F* = 6.90, *p* < 0.001, *R* square = 0.169, Adjusted *R* Square = 0.145.

Abbreviations: SND, secondary nursing diploma; TND, technical nursing diploma.

### 3.2. Qualitative Data Results

The qualitative findings presented below are based on 14 completed and analyzed semistructured interviews.

#### 3.2.1. General Overview of Participants’ Sociodemographic Profile

The dataset includes qualitative responses from 14 nurses:

• Gender: All participants were female.
• Age and ICU‐specific clinical experience: Participants’ ICU‐specific clinical experience ranged from 2 to 23 years. All included participants met the eligibility criterion of having at least 2 years of full‐time ICU‐specific clinical experience.
• Education level: Most held a bachelor’s degree in nursing; a few had master’s degrees, and one had a SND.
• Marital status: Both single and married nurses were represented.
• Work environment: Nurses worked in urban and rural healthcare settings.
• Shifts: Included both day and rotating shifts.
• AI familiarity and use frequency: Interview accounts indicated routine or regular use for some nurses and intermittent or minimal exposure to others. Reported technologies included EHR alerts and monitoring or decision‐support functions, as well as barcode medication administration and smart‐pump safeguards. The latter two were treated as digital safety technologies rather than AI unless linked to predictive, adaptive, or algorithmic decision support. Because no standardized daily or weekly frequency item or system log was collected, these categories are descriptive and should not be interpreted as numerical exposure estimates.

#### 3.2.2. Themes and Subthemes

##### 3.2.2.1. Theme 1: Perceived Support for Clinical Decision‐Making

###### 3.2.2.1.1. Subtheme 1.1: Increased Confidence and Autonomy

Most nurses described AI‐related tools as supporting their clinical decision‐making by validating assessments, drawing attention to alerts, or organizing clinical information. These accounts reflect participants’ perceptions and do not demonstrate an objective improvement in decision quality.

“AI significantly impacts confidence and autonomy…reducing diagnostic errors and increasing confidence in decision‐making.” “It helps, not decides…I still rely on my experience.” These quotations reflect participants’ perceptions and should not be interpreted as evidence of a measured effect.

###### 3.2.2.1.2. Subtheme 1.2: Evidence‐Based and Timely Support

Participants described clinical recommendations and real‐time alerts as useful sources of timely information. They felt that these functions could help them review options more quickly while leaving the final decision with the nurse.

“AI analyzes vast amounts of medical data…offering recommendations based on guidelines.”

“Sometimes it reminds me of something I might miss.”

Number of nurses endorsing this theme: 13 out of 14.

##### 3.2.2.2. Theme 2: Perceived Contributions to Reducing MNC

###### 3.2.2.2.1. Subtheme 2.1: Alert Systems and Early Intervention

Nurses described AI‐enabled and conventional digital safety functions, such as predictive monitor alerts, barcode medication administration, and smart‐pump safeguards, as reminders that could identify potentially dangerous conditions and prompt intervention before complications arose.

“The intelligent IV pump notified me about a dose mistake and prevented the delivery of the dose to the patient. Monitors alert when you are in distress of breathing, these are lifesaving.”

###### 3.2.2.2.2. Subtheme 2.2: Workflow Efficiency

Participants felt that documentation support and automation of repetitive tasks could free time for direct patient care and might reduce delays or omissions. These statements describe perceived possibilities rather than measured effects.

“AI saves me time on routine activities allowing me to pay increased attention to the patient. EHR alerts prevent errors such as inappropriate doses of drugs or allergies.”

Number of nurses endorsing this theme: 12 out of 14.

##### 3.2.2.3. Theme 3: Perceived Support for Communication and Team Collaboration

###### 3.2.2.3.1. Subtheme 3.1: Interprofessional Communication

Participants said that shared access to patient information and structured records could support communication among nurses and other clinicians. One nurse explained, “Communication is enhanced through AI…everybody will see the clear and accurate information. It enhances collaboration and continuity of care in the team.”

###### 3.2.2.3.2. Subtheme 3.2: Shift Handover and Case Coordination

Nurses also felt that structured reports could make handovers clearer and help teams work from the same up‐to‐date information.

“It assists in bettering the system of handoffs and offers structured reports.”

Number of nurses endorsing this theme: 11 out of 14.

##### 3.2.2.4. Theme 4: Humanistic and Ethical Considerations

###### 3.2.2.4.1. Subtheme 4.1: Lack of Emotional and Ethical Intelligence

Numerous nurses stressed that AI does not have emotional intelligence, empathy, and moral reasoning, which are at the core of holistic nursing care.

“The AI is unable to act morally and support the patient psychologically. It will not be able to substitute the human values of nursing such as empathy.”

###### 3.2.2.4.2. Subtheme 4.2: Distrust and Overdependence Risk

Some participants expressed concern that overreliance on AI could diminish critical thinking and clinical judgment, especially when AI outputs are inaccurate or contextually inappropriate.

“Sometimes I suspect AI information – it could be wrong.”

“I use AI as a helpful tool, not a replacement.”

Number of nurses endorsing this theme: 10 out of 14.

##### 3.2.2.5. Theme 5: Professional Learning and Competence

###### 3.2.2.5.1. Subtheme 5.1: Continuous Learning

Participants viewed AI‐related tools as possible aids to learning because they could provide reminders and real‐time feedback. They did not describe these tools as a replacement for formal education or professional judgment.

“It helps me update my information and stay attentive.”

“It promotes continuous learning by showing real‐time feedback.”

###### 3.2.2.5.2. Subtheme 5.2: Gaps in Familiarity and Training Needs

Nurses with less exposure or lower educational levels reported uncertainty or hesitancy toward AI, pointing to the need for structured training and digital competence development.

“I don’t know much about AI…but if it helps, I’d like to learn.”

“I rely more on my experience because I don’t understand the machines well.”

Number of nurses endorsing this theme: 9 out of 14.

#### 3.2.3. Mixed‐Methods Integration of Quantitative and Qualitative Findings

Table 4 presents the joint display summarizing each adjusted quantitative finding alongside the corresponding qualitative evidence and the integrated meta‐inference.

**TABLE 4:** Joint display of quantitative findings, qualitative evidence, and mixed‐methods meta‐inferences.

Quantitative finding	Qualitative evidence	Integrated meta‐inference
Clinical decision‐making: beta = −0.478, *p* < 0.001	Themes 1 and 2: nurses described decision validation, real‐time alerts, timely evidence, early intervention, and workflow support.	Convergence: AI‐supported functions may relate to lower MNC chiefly by strengthening prioritization and timely clinical decisions. The cross‐sectional design does not establish causality.
No AI support or training: beta = 0.175, *p* = 0.007	Theme 5: nurses with less familiarity described hesitation and a need for structured training and continuing support.	Convergence: potential benefits depend on digital competence, implementation support, and continued clinical oversight.
ICU experience: beta = 0.210, *p* < 0.001	No qualitative theme directly explained this coefficient; workflow and coordination accounts offered possible contextual clues.	Expansion: senior‐role demands, case complexity, or reporting awareness may be relevant, but these mechanisms were not measured, and the interpretation remains tentative.
Education level: beta = −0.132, *p* = 0.020	Theme 5 linked lower familiarity and uncertainty with training needs and showed that participants valued continuous learning.	Complementarity: formal education and practical AI familiarity may jointly support safer decisions, but the mechanism requires direct testing.
MISSCARE Reasons: *p* = 0.793 after adjustment	Themes 2 and 3 identified workflow, medication‐safety, handover, and communication mechanisms that participants considered relevant to omissions.	Complementarity: contextual barriers remained experientially salient even though their composite score added no independent linear prediction in the simultaneous model.

The two strands were integrated by comparing each adjusted quantitative finding with the most relevant qualitative theme and identifying convergence, complementarity, or expansion. The small bivariate association between NDMI‐R score and MNC was not statistically significant after adjustment (*β* = −0.124, *p* = 0.063). Nurses nevertheless described alerts, information review, and decision validation as helpful in practice. Their accounts therefore add context but do not establish an AI ⟶ decision‐making ⟶ MNC pathway. The nonsignificant training/support coefficient (*p* = 0.082) was complemented by qualitative requests for structured training. The positive association between experience and MNC (*β* = 0.198, *p* = 0.004) was not directly explained by a distinct theme, so workload or role‐related explanations remain tentative. Likewise, the nonsignificant MISSCARE Reasons coefficient (*p* = 0.057) does not negate participants’ accounts of resource and communication barriers; qualitative salience and adjusted statistical prediction answer different questions.

## 4. Discussion

This study brought together survey findings and nurses’ accounts of AI‐related tools in ICU practice. The quantitative model explained a modest proportion of variation in MNC. Years of experience and the bachelor’s‐or‐above education contrast were statistically significant, whereas the NDMI‐R score, AI training/support indicator, SND contrast, and MISSCARE Reasons composite were not significant after adjustment. These results are associations from cross‐sectional self‐report data and should not be interpreted as effects of AI.

The NDMI‐R finding requires particular care. Higher NDMI‐R scores indicate a more intuitive decision‐making orientation, not better decision quality. The score had a small negative bivariate correlation with MNC, but its adjusted coefficient did not reach statistical significance (*β* = −0.124, *p* = 0.063). Research on missed care suggests that prioritization and situational judgment matter in pressured environments [19–21], but the present data do not show that one decision‐making orientation protects against MNC or mediates an effect of AI.

Years of experience was positively associated with MNC. This differs from evidence that less‐experienced nurses may report more missed basic care [22]. One possible explanation is that experienced ICU nurses are assigned more complex patients, coordinate busy shifts, or carry informal leadership responsibilities. Another is that they may recognize and report omissions more readily. These possibilities were not measured and therefore remain hypotheses for future research rather than explanations established by this study.

Nurses with a bachelor’s degree or above reported lower MNC than the TND reference group, while the SND contrast was not significant. The finding may reflect differences in preparation, role expectations, or access to evidence‐based practice, but it should be interpreted cautiously because educational groups were not randomized and unmeasured workplace factors may contribute. Previous research has not been uniform on the relationship between education and missed care [23].

Although the training/support indicator was not statistically significant in the adjusted model (*p* = 0.082), interview participants repeatedly asked for practical training and ongoing support. They described AI‐related tools as useful when alerts were understandable, information was timely, and the technology fitted their workflow. Similar studies have identified perceived benefit, organizational support, infrastructure, and training as important conditions for AI adoption in nursing [24–26]. The qualitative findings therefore identify implementation priorities without implying that training was a proven predictor of MNC in this dataset.

Participants also linked digital alerts, documentation support, and structured reports with earlier attention to risk, more time for direct care, and clearer handovers. Some examples involved conventional safety technologies, such as barcode medication administration or smart‐pump safeguards, rather than AI itself. We have therefore described these accounts as perceived support from AI‐related or digital tools, not as evidence that AI prevented errors or reduced missed care. This distinction is consistent with the study’s self‐reported exposure measure and the absence of system logs.

The interviews also highlighted limits that matter for responsible implementation. Nurses emphasized that empathy, moral reasoning, and context‐sensitive judgment remain human responsibilities. They were concerned about inaccurate outputs, overdependence, and possible loss of critical‐thinking skills. These concerns echo wider discussions of trust, accountability, and the nurse–patient relationship [27, 28]. AI‐supported CDS should therefore be introduced as an aid to professional judgment, with clear escalation routes and human oversight.

Taken together, the mixed‐methods findings offer a cautious meta‐inference. Nurses perceived AI‐related tools as potentially helpful for information review, workflow, and communication, but the quantitative data did not demonstrate an independent AI effect or a statistically significant adjusted relationship between decision‐making orientation and MNC. Longitudinal studies using verified system‐exposure data, workload and staffing measures, and prespecified analytic models are needed to test the pathways suggested by participants’ accounts.

## 5. Conclusion

ICU nurses described AI‐related decision support as potentially useful for alerts, information review, documentation, and communication. They also stressed that trust, training, human oversight, empathy, and professional judgment must remain central. Quantitatively, the final model explained 16.9% of the variance in MNC. Years of experience and bachelor’s‐level‐or‐higher education, compared with a TND, were significant predictors; decision‐making orientation, training/support, the SND contrast, and MISSCARE Reasons were not statistically significant after adjustment.

The findings do not establish that AI improves decision quality or reduces missed care. Instead, they identify nurses’ experiences and several associations that can guide more rigorous future evaluation. Studies with longitudinal designs, verified measures of AI exposure, and direct measures of workload, staffing, and system performance are needed before causal or implementation‐effectiveness conclusions can be drawn.

### 5.1. Implications

Nursing managers considering AI‐supported CDS should use a staged, nurse‐centered approach. Training should be tied to the specific system and clinical role, and nurses should know when to question an output, override it, or escalate a concern. Leaders should also monitor usability, alert burden, workflow fit, equity, and possible overreliance while preserving accountability for clinical decisions.

These recommendations arise mainly from nurses’ reported experiences and the wider implementation literature, not from a demonstrated effect of AI in this study. Adequate staffing, material resources, and clear communication remain essential. Future evaluations should assess whether a defined AI system changes decision processes or missed care over time and should include verified use data and patient‐safety outcomes.

### 5.2. Strengths and Limitations

Strengths of this study include its convergent mixed‐methods design, use of established instruments, Arabic translation and pilot procedures, reporting guided by STROBE and COREQ, and recruitment across six hospitals. Integrating the two strands allowed the survey findings to be considered alongside nurses’ explanations, including areas where the strands did not align.

Several limitations should be considered. Convenience sampling may have introduced selection bias, and the qualitative sample came from one governorate and included only women, which limits transferability. The quantitative sample was predominantly, but not entirely, female. The cross‐sectional design prevents causal inference, and all primary measures were self‐reported, creating possible recall and social‐desirability bias.

AI exposure was also self‐reported. The study did not collect vendor or software‐version details, system‐use logs, or a standardized frequency measure, so exposure could only be described by reported function and broad qualitative frequency. Some participant examples involved conventional digital safety tools and should not be assumed to be AI‐enabled. The study also did not directly measure workload, burnout, staffing intensity, technological resistance, or informal leadership responsibilities. These unmeasured factors may be relevant to the observed association between experience and MNC.

Finally, translation may not capture every cultural nuance, and AI‐supported systems are changing rapidly. Future multicenter studies should use more gender‐diverse samples, verified measures of system exposure, longitudinal designs, and prespecified models that include workplace and implementation factors.

## Author Contributions

Conceptualization, Faisal Khalaf Alanazi, Ahmed Farghaly Tawfik, and Shimaa Abdelfattah Mahgoub; data curation, Mohamed Hussein Ramadan Atta and Alshaimaa Yehia Mohammed Yehia; formal analysis, Ahmed Farghaly Tawfik and Mohamed Hussein Ramadan Atta; investigation, Shimaa Abdelfattah Mahgoub, Ayman Mohamed El‐Ashry, and Alshaimaa Yehia Mohammed Yehia; methodology, Ayman Mohamed El‐Ashry; supervision, Ahmed Farghaly Tawfik and Ibrahim Naif Alenezi; validation, Ahmed Farghaly Tawfik; writing–original draft, Shimaa Abdelfattah Mahgoub, Mohamed Hussein Ramadan Atta, and Ayman Mohamed El‐Ashry; writing–review and editing, Faisal Khalaf Alanazi, Ibrahim Naif Alenezi, and Enas Mohamed Lotfy.

## Funding

This research was funded by the Deanship of Scientific Research at Northern Border University, Arar, Saudi Arabia (project number NBU‐FPEJ‐2026‐3539‐02).

## Disclosure

All authors have read and agreed to the published version of the manuscript.

## Ethics Statement

Every technique used in this research followed the guidelines and standards outlined in the Declaration of Helsinki (DoH‐Oct2008). Ethical approval for this study was obtained from the Research Ethics Committee of Beni Suef University, Egypt (Reference No. FNBSURECP/2811202416). The research adhered to the ethical guidelines of the Helsinki Declaration, with all participants receiving a detailed explanation of the study’s objectives and procedures. Voluntary participation, anonymity, and confidentiality were strictly maintained throughout the process. Participants were also informed of their right to withdraw at any stage without any negative consequences.

## Consent

Informed consent was obtained from all participants involved in the study.

## Conflicts of Interest

The authors declare no conflicts of interest.

## Data Availability

Data are available from the corresponding author upon reasonable request.
